# Association of Polygenic Variants with Type 2 Diabetes Risk and Their Interaction with Lifestyles in Asians

**DOI:** 10.3390/nu14153222

**Published:** 2022-08-06

**Authors:** Haeng Jeon Hur, Hye Jeong Yang, Min Jung Kim, Kyun-Hee Lee, Myung-Sunny Kim, Sunmin Park

**Affiliations:** 1 Research Group of Personalized Diet, Korea Food Research Institute, Wanju-gun 55365, Jeollabuk-do, Korea; 2 Department of Food Biotechnology, Korea University of Science & Technology, Wanju-gun 55365, Jeollabuk-do, Korea; 3 Department of Food and Nutrition, Obesity/Diabetes Research Center, Hoseo University, 165 Sechul-Ri, BaeBang-Yup, Asan-si 31499, Chungnam-do, Korea

**Keywords:** hyperglycemia, pancreatic β-cell mass, insulin secretion, polygenetic variants

## Abstract

Over the last several decades, there has been a considerable growth in type 2 diabetes (T2DM) in Asians. A pathophysiological mechanism in Asian T2DM is closely linked to low insulin secretion, β-cell mass, and inability to compensate for insulin resistance. We hypothesized that genetic variants associated with lower β-cell mass and function and their combination with unhealthy lifestyle factors significantly raise T2DM risk among Asians. This hypothesis was explored with participants aged over 40. Participants were categorized into T2DM (case; n = 5383) and control (n = 53,318) groups. The genetic variants associated with a higher risk of T2DM were selected from a genome-wide association study in a city hospital-based cohort, and they were confirmed with a replicate study in Ansan/Ansung plus rural cohorts. The interacted genetic variants were identified with generalized multifactor dimensionality reduction analysis, and the polygenic risk score (PRS)-nutrient interactions were examined. The 8-SNP model was positively associated with T2DM risk by about 10 times, exhibiting a higher association than the 20-SNP model, including all T2DM-linked SNPs with *p* < 5 × 10^−6^. The SNPs in the models were primarily involved in pancreatic β-cell growth and survival. The PRS of the 8-SNP model interacted with three lifestyle factors: energy intake based on the estimated energy requirement (EER), Western-style diet (WSD), and smoking status. Fasting serum glucose concentrations were much higher in the participants with High-PRS in rather low EER intake and high-WSD compared to the High-EER and Low-WSD, respectively. They were shown to be higher in the participants with High-PRS in smokers than in non-smokers. In conclusion, the genetic impact of T2DM risk was mainly involved with regulating pancreatic β-cell mass and function, and the PRS interacted with lifestyles. These results highlight the interaction between genetic impacts and lifestyles in precision nutrition.

## 1. Introduction

Type 2 diabetes mellitus (T2DM) is a chronic metabolic disease characterized by the presence of elevated levels of glucose in the blood. A combination of two factors is involved in the T2DM etiology: inadequate insulin secretion by pancreatic β-cells and the inability of insulin-sensitive tissues to respond to insulin, namely, insulin resistance [1]. The etiology of type 2 diabetes is somewhat different in Caucasians and Asians [1]. In a high insulin-resistant state, Caucasians induce hyperinsulinemia to delay T2DM development, but Asians are susceptible to T2DM development due to low insulin secretion with small amounts of islets [2]. The low β-cell mass and function may be associated with antecedent lifestyles, and Asian antecedents were insulin-sensitive due to high grains and fiber intake and good physical activity not to secrete high insulin. However, significant lifestyle changes, including drastic shifts in diet and a more sedentary life over the last three decades, are believed to have led to elevated insulin resistance, which markedly increases the T2DM risk in Asians [1]. Therefore, the rapid increase in insulin resistance due to Westernized lifestyles remarkably elevates T2DM incidence in Asians.

The ethnic differences in the development of T2DM are also linked to the interaction of genetic and environmental factors. Genome-wide association studies (GWAS) have demonstrated that some genetic variants are involved in T2DM in Caucasians and Asians, and the genes linked to T2DM are similar [3,4]. However, Asian and European populations exhibit substantial differences in the allele frequencies of *IGF2BP2, CDKAL1, JAZF1, SCL30A8, HHEX, TCF7L2, EXT2*, and *FTO* [4]. The biological actions of their genetic variants are similar in different ethnic populations, and the ethnic differences in genetic variation are due to the substantial differences in the allele frequencies of the genes related to T2DM [5]. Therefore, genetic differences also need to be considered for formulating therapeutic strategies for T2DM in Asians. Increasing insulin secretion, preserving β-cell mass, and improving glucose utilization by peripheral tissues are the target outcomes for treatment, and the interaction of the genetic variants and lifestyles should be considered for the therapy.

In addition to the genetic differences, food and nutrient intake affect T2DM. The relationship between carbohydrates, fat, protein intake, and T2DM varies with the proportions of their intake, food sources, and cooking types. However, genetics play an essential role in the metabolism of these macronutrients. For example, a high carbohydrate diet is associated with a higher risk of metabolic syndrome, especially hypertriglyceridemia, but not hyperglycemia, in Korean adults [6,7]. It is shown to be related to the interaction of the APOA5, *EFCAB4B*, *ZNF259,* and *APOBEC1* genetic variants with a diet [8,9]. However, plasma vitamin C concentrations and dietary vitamin C intake, but not dietary fiber, improve hyperglycemia in Asian and European populations [10,11]. Therefore, the interaction of the lifestyle factors with genetic variants related to T2DM needs to be studied in each ethnic group.

Genetic variation studies have been conducted on relatively small populations, especially Asians. More studies need to be conducted to explore the impacts of genetic variation on the T2DM etiology and the interactions of the genetic variants with lifestyle. The paucity of large, well-controlled studies of the interactive effects of genetic variants among Asians with diet and lifestyle limits the ability to develop precision medicine treatments for diabetes. However, a few studies have been conducted about the genetic variant interactions with lifestyles. The present study aimed to investigate the genetic variants associated with T2DM and their interaction with lifestyle factors in large hospital-based cohorts of Asians. The results of this study can provide the potential etiological basis for the development of T2DM and its interaction with lifestyles. These results can be applied to a precision medicine approach to the prevention and treatment of T2DM after having been confirmed by dietary intervention trials.

## 2. Materials and Methods

### 2.1. Subjects

Participants over 40 years who had volunteered in a large city hospital-based cohort as part of the Korean Genome and Epidemiology Study (KoGES) conducted during 2010–2014 were included in the study [12]. The institutional review board (IRB) of the Korean National Institute of Health and Hoseo University approved the KoGES (KBP-2015-055) and the present study (HR-034-01). All participants signed a written informed consent form. Because the participants with severe disease states were not included in KoGES, all participants were included for the present study. The parameters influencing T2DM were adjusted for the analysis.

### 2.2. Demographic, Anthropometric, and Biochemical Parameters of the Participants

At the initial visit, demographic parameters including age, gender, education, income, and place of residence were obtained using survey questionnaires. The height and weight were measured with the participant wearing a light gown and bare feet using a well-calibrated digital weight and height scale (Inbody, Cheonan, Korea) [13,14]. Body mass index (BMI) was calculated with the equation of dividing body weight (kg) by the square of height (m^2^). Waist circumferences were determined by taking around the abdomen at the position of two finger-widths above the umbilicus in a relaxing state with a tape measure (Stanley, New Britain, CT, USA). The appendicular skeletal muscle and fat mass were calculated using a machine learning prediction model from the Ansan/Ansung cohort [15].

Blood was collected after more than 12 h fasting in a blood collecting tube with and without heparin and ethylenediaminetetraacetic acid (EDTA). The fasting plasma glucose and HbA1c concentrations were assessed using a Hitachi 7600 Automatic Analyzer (Hitachi, Tokyo, Japan) and an automatic analyzer (ZEUS 9.9; Takeda, Tokyo, Japan). Insulin resistance was estimated using the prediction model generated by the homeostatic model assessment of insulin resistance (HOMA-IR) from a previous study [16].

The frequencies and amounts of alcohol, coffee consumption, physical activity, and smoking history were collected during a health interview. The daily alcohol intake (g/day) was calculated by multiplying the consumption frequencies and amounts [17]. The smoking status was classified into current smokers (at least 20 cigarettes in the past six months), past smokers (no smoking for at least six months, although more than 20 cigarettes in the lifetime), and never been a smoker in the lifetime [17]. Weekly coffee intake was assessed by multiplying drinking frequencies by the amount and was categorized into three groups by tertiles. Regular exercise was defined as more than 30 min of moderate physical activity for three or more days per week.

### 2.3. T2DM Definition

According to the diagnosis guidelines of the American Diabetes Association [18], the participants who had ≥126 mmol/L fasting plasma glucose level, ≥6.5% HbA1c, or were currently using anti-diabetic medication were considered cases of T2DM (case; n = 5383), and the other participants comprised the control (n = 53,318). They were not T1DM or MODY and developed T2DM over age 25.

### 2.4. Estimation of Usual Food Intake Using a Semi-Quantitative Food Frequency Questionnaire (SQFFQ)

The usual food intake during the last 12 months was estimated for each participant using an SQFFQ designed for Korean diet patterns, and its reproducibility and accuracy were validated with three-day food records during the four Korean seasons [19]. The SQFFQ was composed of 106 food items that Koreans commonly consume, and the food frequencies were classified into never or seldom, once per month, two to three times per month, once or twice weekly, three or four times weekly, five or six times weekly, daily, twice daily, and ≥3 times daily. The food consumed at a meal was scored as more than, equal to, or less than the regular portion size visualized using photographs of the 106 foods. The participants recorded the frequencies and the portion size of the 106 food items in the SQFFQ. The daily food intake (g/day) was estimated by multiplying the median of the weekly consumed frequencies by portion sizes. The daily energy, carbohydrates, fats, proteins, vitamins, and mineral intakes were assessed from the daily food intake using the Can-Pro 2.0 nutrient assessment software designed by the Korean Nutrition Society.

### 2.5. Dietary Patterns by Principal Component Analysis (PCA)

The 106 food items in the SQFFQ were categorized into 30 predefined food groups, as previously described [20]. The dietary patterns were classified using the PCA of the 30 predefined groups based on eigenvalues >1.5, and four dietary patterns satisfied these eigenvalues in a PCA [21]. The patterns from the 30 predefined groups were classified using the orthogonal rotation procedure (varimax), and food groups with ≥0.40 factor-loading values were considered the predominant contributors to the assigned dietary pattern [22]. The foods in each dietary pattern were as provided in Supplemental Table S1. According to the foods included in each group, the dietary patterns were named the Korean balanced diet (KBD), plant-based diet (PBD), Western-style diet (WSD), or rice-main diet (RMD) [23,24].

The dietary inflammatory index (DII) was estimated from the prediction equation by multiplying food and nutrient intakes by their dietary inflammatory weights, as reported previously. The equation included energy, 32 nutrients, four food products, four spices, and caffeine [25]. However, garlic, ginger, saffron, and turmeric were absent in the SQFFQ and excluded from the DII calculation. After multiplying the dietary inflammatory scores of 38 foods and nutrients by daily intake, DII was calculated with the sum of their scores divided by 100.

### 2.6. Genotyping Using a Korean Chip and Quality Control

The Center for Genome Science at the Korea National Institute of Health genotyped the participants in the Ansan/Ansung and city hospital-based cohorts. In brief, the genomic DNA was isolated from whole blood, and genotypes were measured using a Korean Chip (Affymetrix, Santa Clara, CA, USA) designed to examine the disease-related SNPs in Koreans [26]. The genotypes were imputed based on the 1000-genome sequence or the Korean HapMap data [27]. The genotyping accuracy was estimated using a Bayesian learning algorithm for Robust General Linear Models (RGLMs) [28]. The inclusion criteria of the genotyping accuracy, missing genotype call rate, and heterozygosity were ≥98%, <4%, and <30%, respectively, and the data showed no gender bias. The genetic variants were included to satisfy the criteria of *p* > 0.05 of the Hardy–Weinberg equilibrium (HWE) and minor allele frequency (MAF) > 5% [28]. Manhattan and QQ plots indicated the accuracy of the GWAS data using the “fastman” library in the R program. A Manhattan plot of the genetic variants was displayed with the negative logarithms of the association *p*-value for T2DM. A QQ plot is a probability plot to show the goodness of fit of the actual data distribution to the theoretical data distribution. The QQ plot of the genotype data displayed the quantile distribution of observed *p*-values (on the *y*-axis) versus the quantile distribution of expected *p*-values (on the *x*-axis). The QQ plot determined the quality of genotypes from the GWAS. When the lambda value of the QQ plot was close to 1, the genotypes by GWAS were ideal. The pathway linked to the genetic variants associated with T2DM with P for Bonferroni correction <0.05 were selected using the MAGMA gene-set analysis in the SNP2GENE of the FUMA web application, available through the git repository (https://github.com/Kyoko-wtnb/FUMA-webapp/, accessed on 8 March 2022).

### 2.7. Genotype-Tissue Expression (GTEx) of Genetic Mutations and Distribution of Identified Tissue/Organ-Specific Expressed SNPs

Genotype-Tissue Expression (GTEx) of genetic mutations uses the source code of the FUMA web application. Normalized gene expression with reads per kilobase of transcript per million reads mapped (RPKM) for 53 tissue types was obtained from GTEx. A total of 56,320 genes were available in GTEx, and we filtered for each tissue with an average RPKM greater than or equal to 1 in at least one tissue type.

In GENE2FUNC, a heatmap of prioritized genes showed two expression values; (i) average log^2^ (RPKM + 1) per tissue per gene where RPKM was Winsorized at 50, allowing a comparison of expression levels between genes and tissue types, and (ii) the mean of the normalized expression (zero mean of log^2^ (RPKM + 1)) per tissue per gene to allow comparison of the expression levels within tissue types within genes. Genes were tested against these differentially expressed genes (DEG) sets by a hypergeometric test to assess whether preferential genes were overrepresented in the DEG sets for specific tissue types. Hierarchical clustering was performed using the python “scipy” package (using the “average” method).

### 2.8. Selection of the Genetic Variants That Influence the T2DM Risk and the Best Model with SNP-SNP Interactions

**Figure 1:**
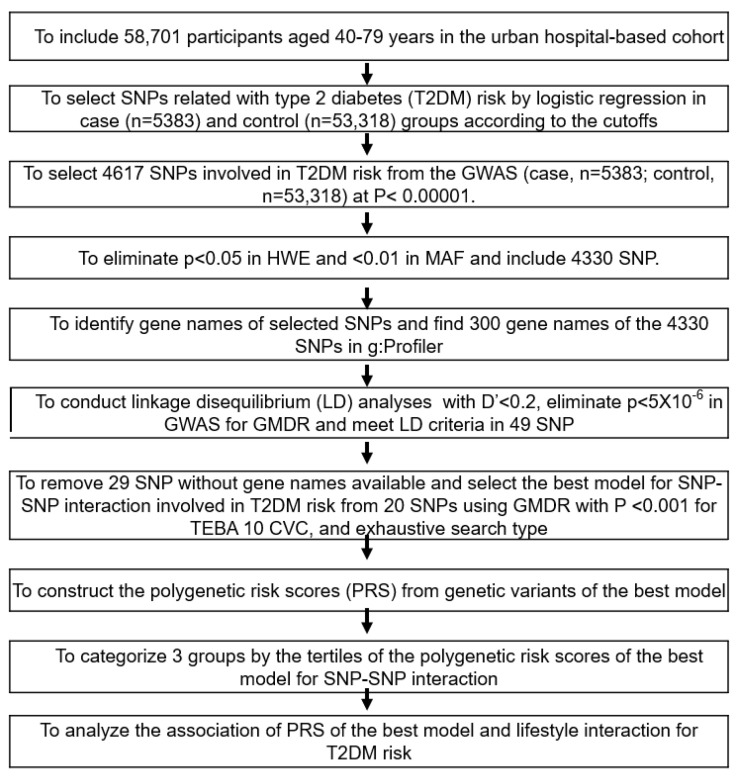
Flow chart for the generation of polygenic risk scores (PRS) to increase the type 2 diabetes risk and interactions between PRS and lifestyles. Type 2 diabetes was defined as ≥126 mmol/L of fasting plasma glucose level, ≥6.5% HbA1c, or currently taking anti-diabetic medication. T2DM (case; n = 5383) and the control (n = 53,318).

Figure 1 represents the best genetic model selection process with SNP-SNP interactions. The GWAS was conducted to explore genetic variants associated with T2DM risk using the T2DM (n = 5383) and control groups (n = 53,318) in the urban hospital-based cohort (*p* < 5 × 10^−5^). Of these, 287 genetic variants did not meet MAF (<5%) and HWE (*p* < 0.05), and the 300 gene names were identified from the 4330 genetic variants using g:Profiler (https://biit.cs.ut.ee/gprofiler/snpense, accessed on 9 February 2022). The SNPs with high D’ values (D’ ≥ 0.2) were excluded because they provided the same information on the genetic impact. Subsequently, 89 genetic variants remained to meet the linkage disequilibrium (LD) criteria (D’ < 0.2) using Haploview 4.2 in PLINK. The potential genetic variants in the same chromosome were not strongly correlated (D’ < 0.2). In addition, 43 genetic variants were removed due to the inability to identify gene names. Among the genetic variants, 29 genetic variants, of which gene names were not identified, were eliminated (n = 29).

Ten genetic variants were selected for the best model with SNP-SNP interaction involved in T2DM risk from 20 SNPs in the generalized multifactor dimensionality reduction (GMDR) with *p* < 0.001 for the sign test of testing balanced accuracy (TEBA) and 10 cross-validation consistency (CVC) in the exhaustive search type and adjustment with covariates of age, gender, residence area, education, and income for models 1, plus energy intake, alcohol intake, regular exercise, and smoking status for model 2. Ten-fold cross-validation was used for CVC because the sample size was larger than 1000 [29].

The PRS for the best model was assessed by summing the number of the risk alleles (genetic risk score) from each selected SNP in the best gene–gene interaction model [30,31]. If the risk allele of the genetic variant was A, the genetic scores of “AA”, “AG”, and “GG” were 2, 1, and 0, respectively. The PRS values in the models with three-, eight-, and 20-SNPs were divided into three categories: Low-PRS, Middle-PRS, and High-PRS. The PRS was classified into Low-, Medium-, and High-PRS as 0–3, 4–5, and ≥6, respectively) in the three-SNP model; 2–7, 8–10, and ≥11 in the eight-SNP model; and 10–19, 20–24, and ≥25 in the 20-SNP model (5 × 10^−6^), respectively. Out of the best models to meet the *p*-value of the sign test and CVC, the model with the lowest SNP number (eight-SNP model) was used to interact with the lifestyle parameters.

### 2.9. Statistical Analysis

The statistical analysis was performed using SAS (version 9.3; SAS Institute, Cary, NC, USA). The 58,701 participants (about 10% of prevalence) were sufficient for the sample size to exhibit significance at α = 0.05, β = 0.99, and an odds ratio of 1.05 in the logistic analysis using a G-power calculator. The descriptive statistics for categorical variables were calculated to show the frequency distributions, and their statistical differences with the T2DM were analyzed using the chi-square test. Descriptive statistics of the continuous variables were assessed to show the adjusted means with standard deviations for the covariates. The statistical differences between the genders in the T2DM group were compared using a two-way analysis of covariance (ANCOVA). When ANCOVA was significant, multiple comparisons according to the genders and T2DM groups were conducted using Tukey’s test.

The association of T2DM with metabolic parameters was analyzed using logistic regression analysis after adjustment for covariates. The results are shown with the adjusted odds ratios (ORs) and 95% confidence intervals (CI) of each metabolic parameter. Two different models were included according to the covariates. Model 1 included age, residence area, survey year, BMI, education, and income as covariates. Model 2 was calculated with covariates of model 1 plus the energy intakes, physical activity, smoking status, and alcohol and coffee consumption.

Two-way ANCOVA was used to analyze the interactions between T2DM and lifestyle-related parameters after they were categorized into the high or low groups according to the dietary reference intake [24] or 30th percentiles of each variable. The two-way ANCOVA model included the main effects of the T2DM and lifestyle-related parameters, their interaction effect, and covariates. The ORs and 95% CI of T2DM with lifestyle-related parameters were also assessed with logistic regression analysis in the high and low groups of the lifestyle-related parameters. The significant difference in the T2DM proportion was analyzed according to the PRS groups using the χ^2^ test in the low- and high groups of the lifestyle-related parameters.

## 3. Results

### 3.1. Demographic and Lifestyle Characteristics

According to genders, the baseline characteristics of the T2DM and control groups are presented in Table 1 since men (13.0%) exhibited a much higher T2DM incidence than women (7.15%). The participants in the T2DM group were older, less educated, and had a lower income than those in the control group of both genders (Table 1). The T2DM risk increased with participants aged over 55 years by 1.88 times and decreased with those educated ≥high school and earned ≥USD 2000/month by 0.646 and 0.748 times, respectively (Table 1). Patients in the T2DM group had a higher BMI, waist circumference, and fat mass than the control group. These factors raised the risk of T2DM by 1.69, 1.92, and 1.58 times, respectively. However, the skeletal muscle mass index (SMI) showed a relation opposite to BMI and was inversely linked with T2DM by 0.77 times (Table 1). As expected, fasting serum glucose and HbA1c concentrations were higher in the T2DM group than in the control group. The incidence of insulin resistance was higher in the T2DM group than in the control group and raised the risk of T2DM by 58.81 times (Table 1).

Nutrient intake was also related to T2DM risk. Energy intake only in men slightly lowered the T2DM group than in the control group, and all participants had no association between energy intake and T2DM risk (Table 2). Carbohydrate and protein intake did not differ between the T2DM and control groups in both genders (Table 2). Fat intake only in women was higher in the T2DM group than in the control group, and it was not associated with T2DM risk. Women’s calcium intake was lower in the T2DM group than in the control group, while vitamin D intake showed the same trend as calcium intake in both genders (Table 2). However, there was no significant difference in the T2DM risk with the intake of these two supplements. Fiber intake did not differ between the T2DM and control groups. Unlike fiber intake, vitamin C and flavonoid intakes were lower in the T2DM group than in the control group, and their intakes were inversely associated with T2DM risk (Table 2). DII was also higher in the T2DM than in the control group, especially in women, and it was positively associated with T2DM risk.

The participants with a high KBD intake were higher in the T2DM group than in the control group in men, but it was the opposite in women. There was no association between the KBD and T2DM risk. PBD was inversely associated with T2DM risk, while WSD was positively associated with it (Table 2). The participants with high PBD intake were lower in the T2DM group than in the control group only for women (Table 2). There was no difference in T2DM incidence with RMD in both genders. Alcohol intake was inversely linked to T2DM risk (Table 2). The participants with moderate exercise were higher in the T2DM group than the control in both genders, and exercise was positively associated with T2DM risk and was related to exercise recommendation to T2DM patients. Smokers were higher in the T2DM group than the control in both genders, and smoking was positively associated with the T2DM risk (Table 2).

### 3.2. Polygenetic Variants with Their Interaction Related to the T2DM Risk

**Figure 2:**
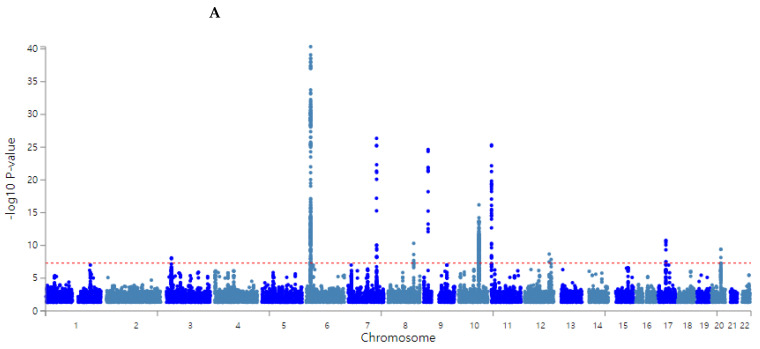

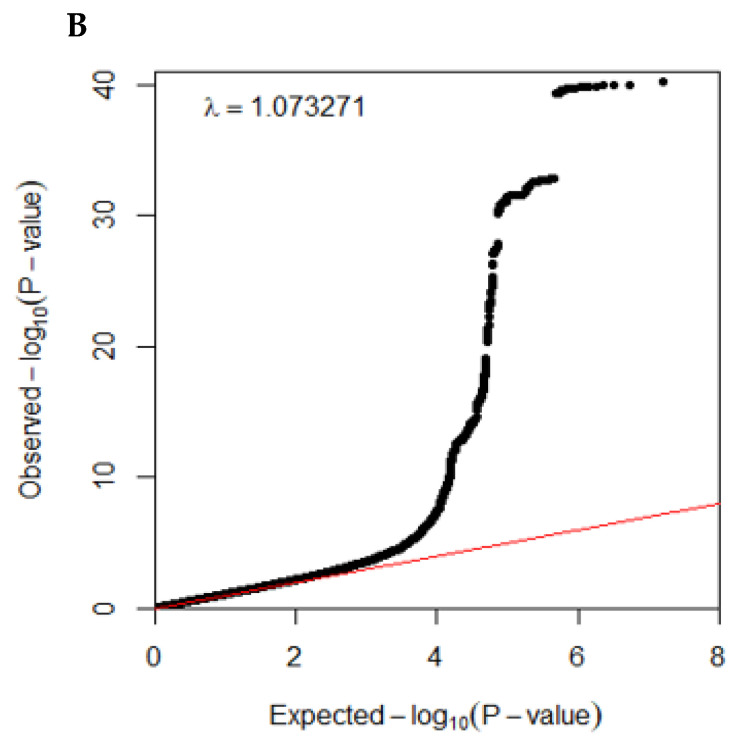
Distribution of genetic variants for type 2 diabetes risk by genome-wide association study: (**A**) Manhattan plot of the *p*-value of genetic variants for type 2 diabetes risk. (**B**) Q–Q plot of observed and expected *p*-values for type 2 diabetes risk by genome-wide association.

The overall statistical association of genetic variants with T2DM is shown as a Manhattan plot (Figure 2A), representing the distribution of genetic variants according to statistical differences. The Q–Q plot shows the quantile distribution of the log of observed *p* values versus the quantile distribution of the log of expected *p* values, and the lambda value, the genome inflation factor, was 1.073, indicating that there was no bias or inflation in the genetic variants from the GWAS for T2DM (Figure 2B).

According to the selection procedure of the genetic variants influencing T2DM risk, the 49 SNPs were selected to satisfy the criteria, including *p* < 5 × 10^−6^ for the GWAS, D’ < 0.2 in LD, *p* ≥ 0.05 in HWE, and ≥0.05 in MAF. Ten genetic variants were selected in the SNP-SNP interaction, and they were rs7631705_*UBE2E2*, rs35612982_*CDKAL1*, rs2191349_ *DGKB*, rs61160304_*PAX4*, rs13266634_*SLC30A8*, rs7034200_*GLIS3*, rs10811661_*CDKN2A/B*, rs12764758_*IDE*, rs60808706_*KCNQ1*, and rs11651052_*HNF1B* (Table 3). The characteristics of genetic variants are listed in Table 3. These ten selected genetic variants were significantly associated with T2DM risk in the city hospital-based and Ansan/Ansung plus rural cohorts at *p* < 5 × 10^−7^ and *p* < 0.05, respectively. The alleles of rs35612982_*CDKAL1*, rs61160304_*PAX4*, rs7034200_*GLIS3*, rs12764758_ *IDE*, and rs11651052_*HNF1B* were positively associated with T2DM risk, while the rest of the alleles were inversely linked to it (Table 3).

**Figure 3:**
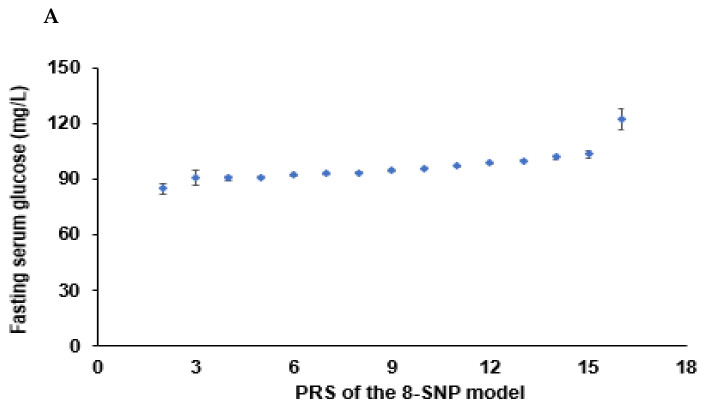

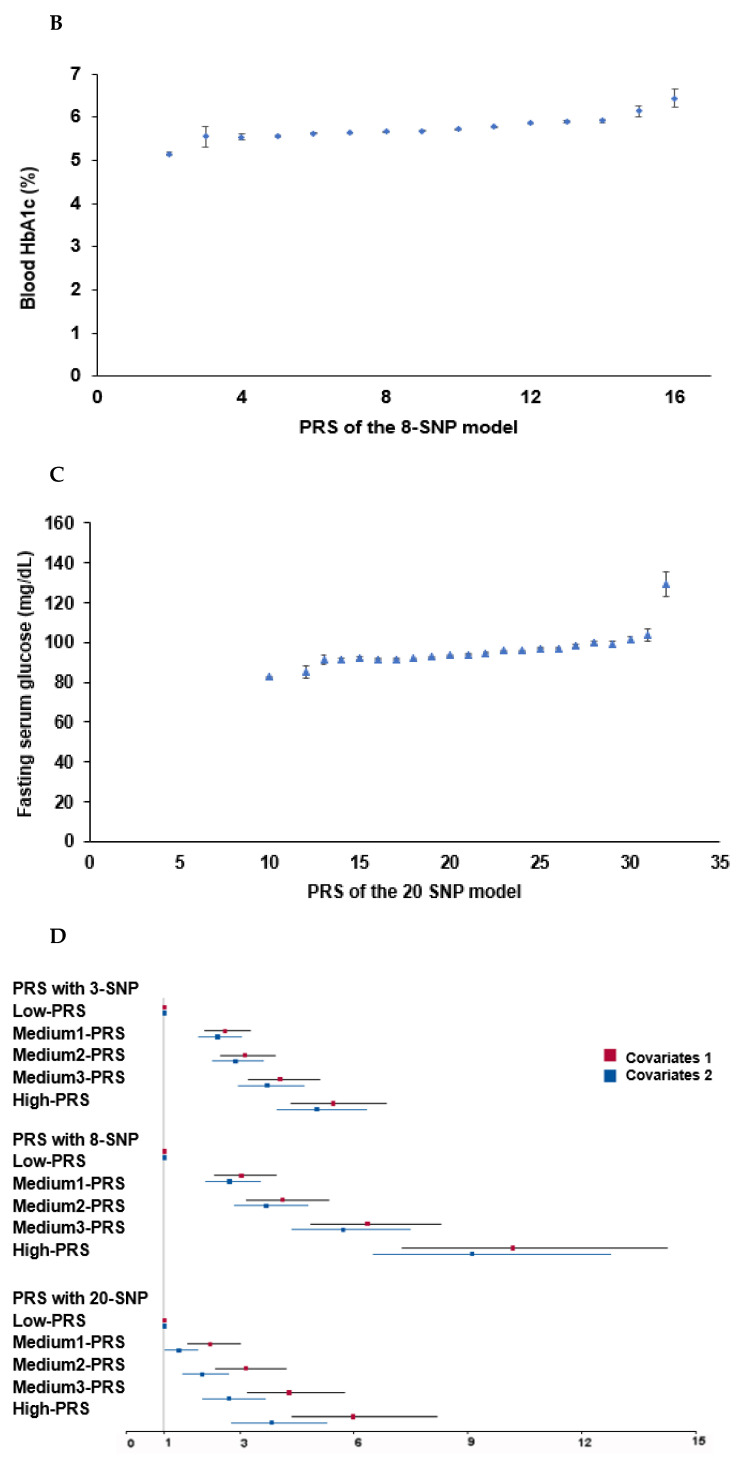
Association of polygenic risk scores (PRS) to type 2 diabetes risk: (**A**) A plot between the PRS of the eight-SNP model and fasting serum glucose concentrations. Dots and bars represented mean and standard errors. (**B**) In a plot between the PRS of the eight-SNP model and blood HbA1c contents, Dots and bars represent mean and standard errors. (**C**) A plot between the PRS of the 20-SNP model and fasting serum glucose concentrations. Dots and bars represented mean and standard errors. (**D**) Adjusted odds ratios (ORs) and 95% confidence intervals (CI) of the PRS of 3- and 8-SNP models. The 3- and 8-SNP models were the models satisfying the criteria of the gene-gene interactions associated with type 2 diabetes risk. The 20-SNP model included all SNPs with *p* < 5 × 10^−8^ in the GWAS of type 2 diabetes risk by adjusting age, gender, education, income, occupation, residence area, and energy intake (percentage of estimated energy requirement) (covariates 1), plus variables in covariate 1, regular exercise, alcohol intake, and smoking status. The PRS was calculated by summing the number of risk alleles of each SNP in the assigned model. The PRS was classified into the Low-, Medium-, and High-PRS groups with 0–3, 4–5, and ≥6 in the three-SNP model; 2–7, 8–10, and ≥11 in the eight-SNP model; and 10–19, 20–24, and ≥25 in the 20-SNP model. Red and blue boxes indicate adjusted ORs for the 3-, 8-, and 20-SNP models, and lines on these boxes indicated 95% confidence intervals.

In the GMDR analysis, models 3, 4, 8, 9, and 10 met the criteria of the sign test (*p* < 0.05) and CVC (10/10), suggesting that they were the candidates for the best models (Table 4). The PRS for the candidate models was calculated, and the association of the PRS with T2DM was determined. The four-SNP model included *CDKAL1*_rs35612982, *CDKN2A/B*_ rs10811661, *KCNQ1*_rs60808706, and *GLIS3*_rs7034200 while the eight-SNP model included four genetic variants plus *UBE2E2*_rs7631705, *HNF1B*_rs11651052, *SLC30A8*_rs13266634, and *PAX4*_rs61160304 (Table 4). The PRS of the four-SNP model was associated with T2DM, 6.1 and 5.8 times in models 1 and 2 with different covariates, respectively, while that of the eight-SNP model was 10.5 and 9.3 times, respectively (Figure 3). The PRS of SNP models 9 and 10 were linked with T2DM by about 6.0 times (data not shown). Furthermore, the PRS of all 20 genetic variants (*p* < 5 × 10^−6^) showed a significant association with T2DM by 6.0 times (Figure 3). In addition, 20 genetic variants with gene names and *p* < 5 × 10^−6^ were found, and the association of their PRS with T2DM was 6.0 times (Figure 3). These results showed that the eight-SNP model was optimal for predicting T2DM risk.

### 3.3. GTEx and Frequency of Tissue/organ-Specific Expression

**Figure 4:**
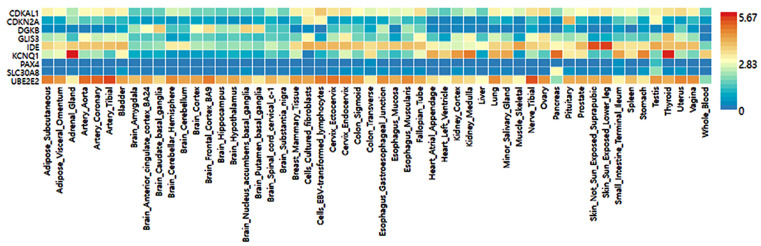
Genotype-Tissue Expression (GTEx) of genes according to their mutation.

The selected genetic variants of the genes influencing T2DM risk were expressed in various tissues, including the brain, adipose tissue, adrenal gland, blood vessel, breast tissue, colon, esophagus, heart, kidney, liver, lung, salivary gland, skeletal muscles, nerve, and ovary (Figure 4). Nine genes of the selected genetic variants were included in the GTEx dataset (Figure 4). In descending order, higher expression of the corresponding gene was seen in the red cells, followed by the blue cells in the risk allele and the non-risk allele in Figure 4. *UBE2E2* was expressed in most tissues but had low expression in the pancreas and blood (Figure 4). The *SLC30A8* risk allele exhibited low expression in most tissues but not in the pancreas. The *KCNQ1* risk allele was observed to have a high expression in the pancreas, thyroid, and adrenal gland compared to the non-risk allele and was relatively highly expressed in the heart and kidney (Figure 4). The *IDE* risk allele was expressed in most tissues but not the brain and heart, while *CDKAL1* was expressed relatively low in most tissues but very low in the brain. *DGKB* and *GLIS3* risk alleles were expressed very low in most tissues (Figure 4).

### 3.4. Metabolism Related to the Genetic Variants for T2DM Risk

A MAGMA gene-set analysis was performed for the curated gene sets and Gene Oncology (GO) terms obtained from MsigDB. Table 5 displays either significant gene sets with a *p*-value of Bonferroni correction or the top 10 genes set when there were greater than ten significant gene sets in GENE2FUNC that were only examined for enrichment of prioritized genes. The genetic variants influencing T2DM from the GWAS were mainly related to the regulation of pancreatic β-cell development, which is linked to maturity-onset diabetes in young adolescents (MODY; Table 5). The β-value represented the cumulative association of genetic variants with the specified pathway. As shown in Table 5, the pathways of the regulation of gene expression in endocrine committed neurog3plus progenitor cells, regulation of β-cell development, and pancreatic endocrine progenitor cells are involved in β-cell development and regeneration. These results suggest that the genetic variants linked to T2DM risk by GWAS were mainly related to β-cell mass regulation, which was also linked to MODY. Moreover, genetic variants related to T2DM risk were associated with the negative regulation of insulin secretion. However, their related genes were not selected in the top 20 SNPs with *p*-values. Therefore, T2DM in Koreans might be genetically close to MODY and mainly related to smaller β-cell mass.

### 3.5. Interaction of PRS with Lifestyle Factors to Influence T2DM Risk

**Figure 5:**
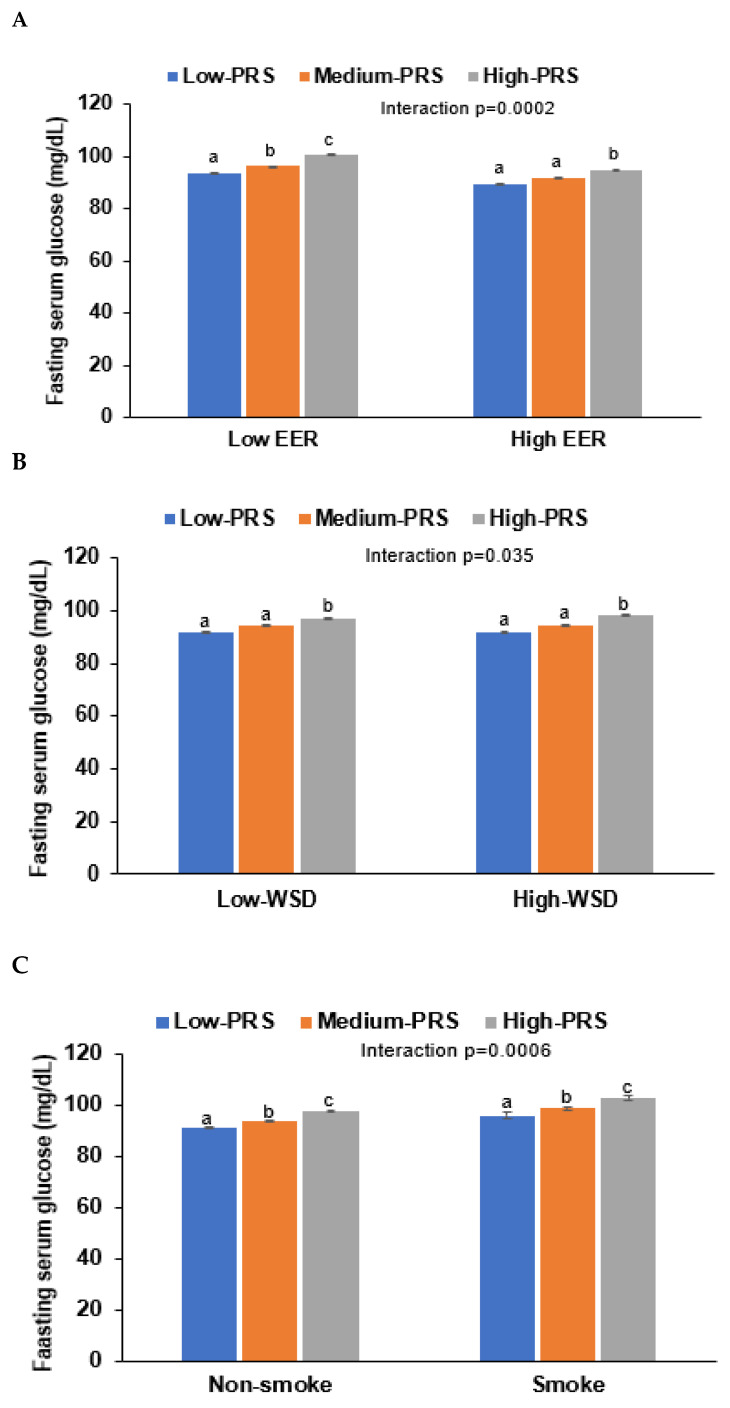
Fasting serum glucose concentrations of participants with low-, medium-, or high-polygenic risk scores (PRS) as determined using the 8-SNP model: (**A**) Adjusted means and standard errors of the serum glucose concentrations according to PRS categories by daily energy intake (a cutoff value: estimated energy requirement, EER). (**B**) Adjusted means and standard errors of the participants according to PRS categories by a Western-style diet (WSD; a cutoff value: 75th percentiles). (**C**) Adjusted means and standard errors of the participants according to PRS categories by smoking status (a cutoff value: smoking). Covariates included age, gender, education, income, energy intake (percentage of estimated energy requirement), occupation, residence area, regular exercise, alcohol intake, and smoking status. ^a,b,c^ Different letters on the bar indicated significant differences among the groups in Tukey’s test at *p* < 0.05.

Energy intake interacted with PRS influencing T2DM risk, and PRS was positively associated with T2DM risk in high and low energy intakes by 3.59 and 2.96 times, respectively (Table 6). Fasting serum glucose concentrations increased in High-PRS compared to Low-PRS in low and high energy intake groups, but interestingly, they were lower in all the PRS groups in the low energy intake groups compared to the high groups (Table 6, Figure 5A). These results may be due to the lower energy intake in T2DM patients.

Only WSD interacted with PRS for T2DM risk in the four dietary patterns, but KBD, PBD, and RMD did not interact with it (*p* < 0.035; Table 6). However, the statistical significance was not large enough to pass the *p*-value of the Bonferroni correction. High and low WSD intakes were positively associated with T2DM risk by 3.13 and 3.33 times, respectively (Table 6). However, fasting serum glucose was lower in the High-WSD group than in the Low-WSD group in all PRS groups (Figure 5B).

In an assessment of lifestyles, alcohol intake and exercise also did not interact with PRS to affect the T2DM risk, but smoking status interacted with it (Table 6). These results suggest that the smoking state affected T2DM risk in the participants with High-PRS. Serum glucose concentrations were much higher in the High-PRS group than in the Low-PRS group, especially in smokers (Figure 5C). Therefore, it was recommended that the participants with High-PRS should not smoke, and low energy intake did not reduce T2DM risk.

## 4. Discussion

The etiology of T2DM in Asians is somewhat different from that of Caucasians [1,2]. T2DM is characterized by increased insulin resistance and β-cell dysfunction. Although both factors are hallmarks of T2DM, recent studies suggest ethnic differences in β-cell function, wherein Asians have less β-cell functional capacity than Caucasians [1,2]. These findings indicate that genetic variants related to β-cell function and mass may differently influence T2DM risk in two populations. The genetic variants related to T2DM in Caucasians are mainly related to insulin resistance [32], but those in Asians, including Chinese, Japanese, and Koreans, are involved in insulin secretion (*GLP1R, PAX4, HNF4A, SLC30A8, HHEX, CDKAL1, CDKN2A/B*, and *GCKR*) [33,34,35,36]. The present study showed that the genetic variants related to T2DM risk were involved in pancreatic β-cell development, growth, and insulin secretion and were linked to MODY genes. The best model for genetic variant–genetic variant interaction included *CDKAL1*_rs35612982, *CDKN2A/B*_rs10811661, *KCNQ1*_ rs60808706, *GLIS3*_rs7034200, *UBE2E2*_rs7631705, *HNF1B*_rs11651052, *SLC30A8*_rs13266634, and *PAX4*_rs61160304. The High-PRS of the eight-SNP model was associated with T2DM by about 10 times. According to genetic variants, the gene expression in the 7-SNP model was not provided in GTEx. However, their gene expression was different in various tissues. Most genes except *IDE* and *UBE2E2* exhibited low expression in most tissues. However, *KCNQ1*, *SLC30A8*, and *IDE* with the risk alleles were relatively highly expressed in the pancreas compared to the non-risk alleles.

MODY is monogenic diabetes caused by a single gene mutation of either *HNF-1α*, *HNF-1β*, *HNF-4α*, *HNF-1β*, glucokinase, *PAK4*, *KLF11*, neurogenic differentiation 1 (*neuroD1*), and insulin (*INS*). MODY is typically developed before the age of 25 [37,38]. MODY is linked to the defects in pancreatic islet cell development and insulin secretion in young individuals. MODY is linked to non-obesity, similar to Asian T2DM [38]. The present study demonstrated that Korean T2DM included the mutation of several genes also related to MODY. However, T2DM in this study was not a monogenic disease like MODY, and the participants with T2DM developed diabetes after age 25. Therefore, Asian T2DM was related to low β-cell function and mass, and some of the common genetic variants were in genes also linked to MODY. *PAX4* and *HNF1β* genetic variants were included in the best model, while those of *HNF-1α, HNF-1β,* *KLF11, neuroD1*, and glucokinase, were additionally involved at *p* < 0.00001 [39]. Unlike MODY single gene mutations, the combination of genetic variants of several MODY-related genes induced T2DM in Korean adults. These results suggest that the etiology of Korean T2DM is associated with specific genetic predispositions that impair β-cell function and mass, and this theory may be extended to all Asians.

Furthermore, the genetic variants that raise the risk of T2DM were related to β-cell development and survival and insulin secretion in the present study. The study supports the current evolving data that the higher risk of T2DM in Asians is connected to their smaller pancreatic β-cell mass. The genetic variants involved in the pancreatic β-cell mass through modulating β-cell development and survival result in impaired glucose metabolism to induce T2DM [40]. *KCNQ1* mutations are reported to reduce insulin secretion and decrease pancreatic β-cell mass [41]. Although it remains unclear, *KCNQ1* mutations are believed to increase cyclin-dependent kinase inhibitor 1C (*CDKNL*) through epigenetic modification [42]. Along with MODY genes, *KCNQ1* mutations could influence T2DM by decreasing pancreatic β-cell mass and insulin secretion as per the present study.

*SLC30A8* is also involved in providing zinc for insulin-hexamer formation, and it binds to *PDX-1*, linked to pancreatic β-cell growth. The decrease in zinc transport due to its mutation is associated with T2DM risk [43]. The *PDX-1* mutation can suppress its binding to *SLC30A8*, decreasing β-cell growth to modulate T2DM susceptibility. Glucose-stimulated insulin secretion in the islets from *SLC30A8* knockout mice is also suppressed [43]. *SLC30A8* is involved in β-cell growth, proinsulin modification, and glucose-stimulated insulin secretion. It is a missense mutation to truncating protein (Trp325Arg; rs13266634) carriers that exhibit a 65% reduction in T2DM risk [43], which was consistent with the present study (OR = 0.8529, *p* < 8.22 × 10^−12^). The *SLC30A8* mutation is involved in releasing insulin secretory granules by zinc flux proinsulin modification. The risk allele carrier of the Trp325Arg mutation reduces zinc transporter activity, but its impact on glucose metabolism is varied in observational studies. The mutation exhibits modest hyperglycemia in a high-fat diet.

In addition, *CDKAL1* variants, including rs35612982, are strongly involved in increased T2DM and obesity risk [44]. *CDKAL1* act as a tRNA^Lys^ modifier, and *CDKAL1* loss impairs proinsulin translational fidelity in pancreatic β-cells, contributing to glucose-dependent insulin secretion [44]. Its variants also affect insulin response in persons of European ancestry [45]. *CDKN2A/B* locus encodes p15 and p16 inhibitors for cyclin-dependent kinase 4 (*CDK4*), which regulates pancreatic β-cell replication [46]. The increased expression of *CDKN2B* induces hypoplasia of the exocrine and endocrine glands in rodents, and *CDK4* inhibition induces insulin deficiency due to decreased pancreatic β-cell counts [46]. *CDKN2A/B*_rs10811661 carriers are susceptible to T2DM risk in different ethnic groups, including the Iraqi, Chinese, and Tai populations [46,47,48]. Consistent with the present study results, Asians carrying *CDKAL1*_rs35612982, *CDKN2A/B*_rs10811661, and *KCNQ1*_rs60808706, which are mainly linked to β-cell mass, have an elevated susceptibility to T2DM, suggesting that T2DM in Asians may be closely related to reduced β-cell mass and function.

T2DM risk is linked to genetics and multiple lifestyle factors. Managing this interaction could modulate the risk of developing T2DM in genetically susceptible to T2DM. The PRS of the best model for T2DM interacted with energy intake, but it did not interact with the macronutrient composition in the present study. Previous studies have demonstrated that some of the genetic variants associated with T2DM included in the PRS found in the present study have interactions with nutrients and other lifestyle factors [49]. Furthermore, the PRS interacted with WSD, and in a high WSD intake, high-PRS increased T2DM risk compared to low-PRS in the present study. In a systematic review, Western dietary patterns have interacted with T2DM-related PRS, fat and carbohydrate intake with *IRS1*_rs2943641, and physical activity with *HNF1B*, *IRS1*, *PPARγ*, *ADRA2B*, *SLC2A2*, and *ABCC8* variants in European ethnicities [5,49]. In Asians, PRS related to T2DM interacted with energy and calcium intake [36], and PRS related to insulin secretion interacted with Western-style diets [50]. Asian antecedents were once insulin-sensitive due to high dietary fiber intake and good physical activity [51]. However, significant lifestyle changes, including drastic shifts in diet to WSD and a more sedentary life over the last three decades, are believed to have led to elevated insulin resistance. WSD is characterized by high fat and low fiber diet, which enables acidic conditions in the body [52]. Acidic load, mainly by mineral balance, is reported to damage bone [52], but it may be involved in β-cell dysfunction. Therefore, the results suggest that carriers with a high PRS should be recommended an adequate energy intake with sufficient nutrients but avoid a Western-style diet.

This study has some strengths and limitations. The strength of the current study was to be conducted on a large hospital-based cohort in Korea (n = 58,701). It was also well designed, and specialists collected test samples uniformly from the volunteers. However, it had some limitations: First, it was cross-sectionally conducted, and the results cannot be directly applied to cause-and-effect. Second, daily food intake during the past six months was estimated from the SQFFQ, including 106 food and dishes commonly consumed by Koreans. SQFFQ could not evaluate the exact amounts of each food and dish, but it represented the usual nutrient intake. SQFFQ could be appropriate for estimating the usual nutrient intake in big cohort studies. Third, regular exercise was defined as more than 30 min of moderate physical activity for three or more days per week, which was not validated. Fourth, genetic variants were also determined with a customized K-chip (Axiom Biobank plus Genotyping Array, KNIHv1.1), and it contained tagging SNPs that maximized genomic coverage and functional SNPs such as nonsynonymous, expression quantitative trait loci (eQTL), and previously known reported disease-associated SNPs [53]. Its reproducibility and accuracy were 99.77 and 99.73%, respectively [53], and the K-chip is optimal for exploring the genetic variants for T2DM risk in Koreans.

In conclusion, the participants with a High-PRS of the eight-SNP model that included rs7631705_*UBE2E2*, rs35612982_*CDKAL1*, rs61160304_*PAX4*, rs13266634_*SLC30A8*, rs7034200_*GLIS3*, rs10811661_*CDKN2A/B*, rs60808706_*KCNQ1*, and rs11651052_*HNF1B* showed a 10-fold elevation in T2DM risk compared to those with a Low-PRS. The selected genetic variants were mainly involved with regulating pancreatic β-cell mass and function, and the PRS interacted with lifestyles. This study clearly demonstrated that Asians with a high T2DM-related genetic predisposition are at much greater risk of developing diabetes, and that risk is exacerbated by interaction with a Westernized lifestyle, including diet, sedentary lifestyles, and smoking. PRS interacted with energy intake, WSD, and smoking, indicating that the participants with high PRS generally failed to compensate for high insulin resistance by elevating insulin secretion, especially when having WSD and smoking. Therefore, this study suggests that people with a high PRS may benefit from consuming adequate nutrition to meet EER while decreasing intakes of WSD and avoiding smoking. These results provide a potential genetic mechanism that places individuals at high risk of T2DM and readily interacts with diet and lifestyle to facilitate progression to T2DM. They can be applied in precision nutrition to prevent and alleviate T2DM.

## Figures and Tables

**Table 1:** Demographic and anthropometric characteristics according to genders and type 2 diabetes (T2DM).

	Men (n = 20,293)		Women (n = 38,408)		Adjusted ORs and 95% CI
	Control (n = 17,656)	T2M (n = 2637)	Control (n = 35,662)	T2DM (n = 2746)	
Age (years)	55.7 ± 0.06 ^b^	58.7 ± 0.15 ^b^	52.3 ± 0.04 ^c^	55.5 ± 0.14 ^b^ *** ^+++^	1.875 (1.740–2.022)
Education					
≤Middle school	1467 (13.7)	286 (16.1) ^‡‡^	5887 (21.1)	851 (34.8) ^‡‡‡^	1
High school	8103 (75.7)	1331 (75.1)	20,360 (72.9)	1511 (61.8)	0.723(0.664–0.786)
≥College	1136 (10.6)	155 (8.75)	1698 (6.08)	84 (3.43)	0.646 (0.550–0.760)
Income					
≤USD 2000	1338 (7.96)	268 (10.7) ^‡‡‡^	3677 (11.0)	495 (19.1) ^‡‡‡^	1
USD 2000–4000	7082 (42.1)	1125 (44.9)	14,700(43.8)	1285 (49.7)	0.846 (0.770–0.928)
>USD 4000	8389 (49.9)	1111 (44.4)	15,163(45.2)	807 (31.2)	0.748 (0.676–0.827)
BMI (kg/m^2^)	24.3 ± 0.02 ^b^	25.0 ± 0.06 ^a^	23.5 ± 0.02 ^c^	25.0 ± 0.06 ^a^ *** ^+++ ###^	1.692 (1.557–1.838)
Waist circumferences (cm)	84.2 ± 0.04 ^b^	85.2 ± 0.10 ^a^	78.7 ± 0.03 ^d^	79.8 ± 0.09 ^c^ *** ^+++^	1.916 (1.752–2.095)
SMI (kg/m^2^)	7.33 ± 0.01 ^a^	7.20 ± 0.01 ^b^	6.22 ± 0.01 ^c^	6.06 ± 0.004 ^d^ *** ^+++ ###^	0.768 (0.638–0.924)
Fat mass (%)	23.1 ± 0.03 ^d^	24.0 ± 0.07 ^c^	31.0 ± 0.02 ^b^	32.6 ± 0.07 ^a^ *** ^+++ ###^	1.578 (1.429–1.742)
Serum glucose (mg/dL)	93.3 ± 0.17 ^c^	133.8 ± 0.40 ^a^	90.3 ± 0.12 ^d^	129.1 ± 0.42 ^b^ *** ^+++ ###^	
HbA1c (%)	5.52 ± 0.01 ^b^	7.04 ± 0.02 ^a^	5.56 ± 0.01 ^b^	7.08 ± 0.02 ^a^ ** ^+++^	
Insulin resistance (%)	680 (3.65)	1632 (61.9) ^‡‡‡^	791 (2.22)	1501 (54.7) ^‡‡‡^	58.81 (51.59–67.04)

Values represent adjusted means and standard errors. Values represent adjusted odd ratios (ORs) and 95% confidence intervals (CI). Covariates included age, sex, education, income, energy intake (percentage of estimated energy requirement), residence areas, daily activity, alcohol intake, and smoking status. Skeletal muscle mass index (SMI) was calculated by dividing the limb skeletal muscle mass (kg) by the square of the height (m^2^). The cutoffs of the logistic regression analysis were as follows: 55 years for age, 25 kg/m^2^ for BMI, 90 cm in men and 85 cm in women for waist circumferences, 75th percentiles for SMI, and 25% in men and 32% in women for fat mass. ** Significant differences by genders at *p* < 0.01, *** *p* < 0.001. ^+^^++^ Significant differences by T2DM at *p* < 0.001. ^#^^##^ Significant interaction between genders and obesity at *p* < 0.001. ^‡‡^ Significantly different from the control group in X2 test in each gender at *p* < 0.01, ^‡‡‡^ at *p* < 0.001. ^a,b,c,d^ Different superscripts indicate significant differences among the groups by Tukey test at *p* < 0.05.

**Table 2:** Lifestyles and daily nutrient intake according to genders and T2DM.

	Men (n = 20,293)		Women (n = 38,408)		Adjusted ORs and 95% CI
	Control (n = 17,656)	T2DM (n = 2637)	Control (n = 35,662)	T2DM (n = 2746)	
Energy intake (EER %) ^1^	86.0 ± 0.06 ^1 b^	85.4 ± 0.13 ^c^	104 ± 0.05 ^a^	1040 ± 0.09 ^a^ *** ^###^	0.958 (0.767–1.196)
CHO (En%) ^2^	71.6 ± 0.08	71.3 ± 0.17	71.7 ± 0.06	71.6 ± 0.12	0.997 (0.932–1.067)
Fat (En%) ^3^	13.9 ± 0.06 ^a b^	14.2 ± 0.12 ^a b^	13.9 ± 0.04 ^b^	14.1 ± 0.09 ^a ##^	0.987 (0.893–1.091)
Protein (En%) ^4^	13.3 ± 0.03 ^c^	13.4 ± 0.05 ^b^	13.6 ± 0.02 ^a^	13.6 ± 0.04 ^a^ *** ^+ #^	1.054 (0.978–1.136)
Fiber (g) ^5^	14.3 ± 0.07 ^a^	14.3 ± 0.12 ^a^	14.8 ± 0.07 ^b^	14.9 ± 0.08 ^b^ ***	0.740 (0.266–2.054)
Calcium (mg) ^6^	383 ± 1.61 ^c^	385 ± 4.00 ^c^	475 ± 1.11 ^a^	463 ± 3.91 ^b^ *** ^#^	0.985 (0.906–1.071)
Vitamin C (mg) ^7^	89.3 ± 0.47 ^c^	89.0 ± 1.16 ^c^	114.2 ± 0.32 ^a^	110 ± 1.13 ^b^ *** ^++ #^	0.918 (0.857–0.983)
Vitamin D (ug) ^8^	5.24 ± 0.04 ^c^	4.98 ± 0.08 ^d^	7.18 ± 0.04 ^a^	6.95 ± 0.06 ^b^ *** ^++^	0.967 (0.871–1.075)
DII (scores) ^9^	−18.3 ± 0.12 ^c^	−18.5 ± 0.29 ^c^	−21.6 ± 0.28 ^a^	−20.7 ± 0.08 ^b^ *** ^++^	1.109 (1.028–1.195)
Flavonoids (mg) ^10^	30.0 ± 0.24 ^c^	29.9 ± 0.60 ^d^	43.1 ± 0.17 ^a^	40.0 ± 0.59 ^b^ *** ^+++^	0.891 (0.809–0.981)
KBD (%) ^11^	6671 (39.6)	1430(41.7) ^‡^	9165 (30.1)	2303(28.8) ^‡^	0.958 (0.910–1.008)
PBD (%) ^11^	3488 (20.7)	710 (20.7)	12,346 (40.6)	3032(37.9 ^‡‡‡^	0.890 (0.827–0.958)
WSD (%) ^11^	8489 (50.4)	1933 (56.3) ^‡‡‡^	10,333 (34.0)	2788(34.8)	1.269 (1.207–1.335)
RMD (%) ^11^	5376 (31.9)	1089(31.7)	10,370 (34.1)	2736(34.2)	1.018 (0.970–1.068)
Alcohol (g) ^12^	35.7 ± 0.38 ^a^	36.6 ± 0.94 ^a^	5.37 ± 0.26 ^b^	4.96 ± 0.91 ^b^ ***	0.878 (0.818–0.942)
Exercise (%) ^13^	10,323 (58.7)	1629 (61.9) ^‡‡^	18,537 (52.2)	1487 (54.3) ^‡^	1.143 (1.073–1.217)
Non-smoking (%)	5150 (29.2)	629 (23.9) ^‡‡‡^	34,442 (96.9)	2618 (95.7) ^‡‡‡^	1
Former smoking	7541 (42.8)	1254 (47.7)	427 (1.2)	33 (1.21)	1.289 (1.163–1.430)
Smoking	4919 (27.9)	745 (28.4)	664 (1.87)	85 (3.11)	1.602 (1.431–1.792)

^1^ Values represent adjusted means and standard errors. ^1^ Values represent adjusted odd ratios and 95% confidence intervals. Covariates included age, sex, education, income, energy intake (percentage of estimated energy requirement), residence areas, daily activity, alcohol intake, and smoking status. KBD, Korean balanced diet; PBD, plant-based diet; WSD, Western-style diet; RMD, rice-main diet. The cutoffs of the logistic regression analysis were as follows: ^1^ estimated energy requirement (EER), ^2^ 70 energy percent (En%) for carbohydrate (CHO), ^3^ 15 En% for fat, ^4^ 14 En% for protein, ^5^ 20 g for fiber, ^6^ 500 mg for calcium, ^7^ 100 mg for vitamin C, ^8^ 10 ug for vitamin D, ^9^ −25 scores for dietary inflammatory scores, ^10^ 45 mg for flavonoids, ^11^ 70th percentiles of each dietary pattern, ^12^ 20 g for alcohol, and ^13^ moderate exercise for 150 min/week. *** Significant differences by genders at *p* < 0.001. ^+^ Significant differences by type 2 diabetes (T2DM) at *p* < 0.05, at ^++^ *p*<0.01, ^+++^ *p* < 0.001. ^#^ Significant interaction between genders and obesity at *p* < 0.05, ^##^ at *p* < 0.01, ^###^ *p* < 0.001. ^‡^ Significantly different from the control group in χ^2^ test in each gender at *p* < 0.05, ^‡‡^ at *p* < 0.01, ^‡‡‡^ at *p* < 0.001. ^a,b,c,d^ Different superscripts indicate significant differences among the groups by Tukey test at *p* < 0.05.

**Table 3:** Characteristics of genetic variants selected for their interactions by generalized multifactor dimensionality reduction.

Chr ^1^	SNP ^2^	Position	Mi ^3^	Ma ^4^	OR and 95% CI for City ^5^	*p* Value Adjusted *^6^*	*p* Value Adjusted *^7^*	MAF ^8^	*p* Value for HWE ^9^	Gene	Functional Consequence
3	rs7631705	23632234	C	T	0.888	8.00 × 10^−9^	0.0395	0.3341	0.5715	*UBE2E2*	3_prime_utr
6	rs35612982	20682622	C	T	1.342	9.35 × 10^−39^	1.27 × 10^−8^	0.4649	0.1131	*CDKAL1*	Intron
7	rs2191349	15064309	G	T	0.891	2.91 × 10^−7^	0.0175	0.3233	0.04656	*DGKB*	Intron
7	rs61160304	127249659	T	C	1.492	6.34 × 10^−26^	2.11 × 10^−7^	0.0738	0.2274	*PAX4*	Downstream
8	rs13266634	118184783	T	C	0.853	8.22 × 10^−^^12^	0.00255	0.398	0.9656	*SLC30A8*	Missense
9	rs7034200	4289050	A	C	1.113	2.05 × 10^−7^	0.0150	0.4064	0.3467	*GLIS3*	Nmd transcript
9	rs10811661	22134094	C	T	0.797	6.33 × 10^−24^	1.08 × 10^−6^	0.4387	0.1998	*CDKN2A/B*	Non-coding transcript
10	rs12764758	94516663	T	C	1.285	5.00 × 10^−10^	0.0304	0.0586	0.3958	*IDE*	Intron
11	rs60808706	2857233	A	G	0.787	6.65 × 10^−25^	8.17 × 10^−7^	0.3913	0.2251	*KCNQ1*	Downstream
17	rs11651052	36102381	A	G	1.157	5.17 × 10^−10^	0.008245	0.3009	0.4383	*HNF1B*	Intron

^1^ Chromosome; ^2^ Single nucleotide polymorphism; ^3^ Minor allele; ^4^ Major allele ^5^ Odds ratio (OR) and 95% confidence intervals (CI) for city cohort; ^6^ *p*-value for OR after adjusting for age, gender, residence area, survey year, body mass index, daily energy intake, education, and income in the city cohort; ^7^ *p*-value for OR for Ansan/Ansung cohort; ^8^ Minor allele frequency; ^9^ Hardy–Weinberg equilibrium.

**Table 4:** Generalized multifactor dimensionality reduction (GMDR) results from multi-locus interaction with genes related to β-cell function for type 2 diabetes risk.

Covariates			Adjusted for Age, Gender, BMI, Education, Income, Income, Area				Adjusted for Age, Gender, BMI, Education, Income, Income, Area, Smoke, Exercise, Alcohol, and Energy Intake			
Models			TRBA	TEBA	*p* Value	CVC	TRBA	TEBA	*p* Value	CVC
*CDKAL1*_rs35612982			0.5411	0.5412	0.001	10	0.5411	0.5412	0.001	10
*CDKN2A/B*_rs10811661 plus model 1			0.5529	0.5493	0.001	10	0.5529	0.5493	0.001	10
*KCNQ1*_rs60808706 plus model 2			0.5599	0.5589	0.001	10	0.5599	0.5589	0.001	10
*GLIS3*_rs7034200 plus model 3			0.5665	0.5578	0.001	9	0.5665	0.5578	0.001	9
*UBE2E2*_rs7631705 plus model 4			0.5778	0.5552	0.001	8	0.5778	0.5552	0.001	8
*HNF1B*_rs11651052 plus model 5			0.5985	0.5393	0.001	7	0.5985	0.5393	0.001	7
*SLC30A8*_rs13266634 plus model 6			0.6395	0.5294	0.001	7	0.6395	0.5294	0.001	7
*PAX4*_rs61160304 plus model 7			0.706	0.5208	0.001	10	0.706	0.5208	0.001	10
*IDE*_rs12764758 plus model 8			0.7644	0.5215	0.001	10	0.7644	0.5215	0.001	10
*DGKB*_rs2191349 plus model 9			0.812	0.5218	0.001	10	0.812	0.5218	0.001	10

TRBA, trained balanced accuracy; TEBA, test balance accuracy; CVC, cross-validation consistency; sign test, result and *p* value for the significance of GMDR model by sign test with and without adjusting for covariates designated in the table; BMI, body mass index.

**Table 5:** Pathways related to genetic variants for type 2 diabetes.

Pathways	No. of Genes ^1^	Beta ^2^	SD ^3^	*p* Value ^4^	*p* Value Bonferroni ^5^	Participating Genes
Regulation of gene expression in endocrine committed neurog3plus progenitor cells	2	1.677	0.0229	2.92 × 10^−15^	4.45 × 10^−11^	*PAX6, HNF1α, HNF1β*
Maturity onset diabetes of the young	16	0.6309	0.0243	3.88 × 10^−13^	5.92 × 10^−09^	*PDX1, HNF1β, HNF1a, HNF4α, NeuroD1*
Regulation of β-cell development	24	0.4618	0.0218	8.64 × 10^−10^	1.32 × 10^−05^	*HNF1β, FGF10, ONECUT3, HNF6, PDX1*
Pancreatic endocrine progenitor	6	0.8761	0.0207	1.12 × 10^−09^	1.72 × 10^−05^	
Negative regulation of hormone secretion	34	0.3263	0.0183	6.46 × 10^−08^	9.85 × 10^−04^	
Negative regulation of insulin secretion	22	0.3953	0.0179	1.58 × 10^−07^	0.0024	*ADR2α, CRHR2, KLF7, PDE1c, UCP2*

^1^ The number of genes related to type 2 diabetes risk. ^2^ The resulting coefficient from a fit between genetic variants for type 2 diabetes with the pathway. ^3^ SD, standard deviation of beta; ^4^ *p* value for the beta for type 2 diabetes. ^5^ *p* value with Bonferroni correction for the beta for type 2 diabetes. *PAX6*, paired box protein 6, *HNF*, hepatocyte nuclear factor; *NeuroD1*, neuronal Differentiation 1; *FGF10,* fibroblast growth factor 10; *ONECUT3*, one cut homeobox 3; *PDX1*, pancreatic and duodenal homeobox 1; *ADR2α*, adrenergic α2a; CRHR2, *corticotropin-releasing hormone receptor 2;* *KLF7*, Kruppel-like factor 7, *PDE1c*, Phosphodiesterase 1C; UCP2, Uncoupling Protein 2.

**Table 6:** Adjusted odds ratios for the T2DM risk by polygenetic risk scores of the best model (PRS) for gene–gene interaction after covariate adjustments according to the lifestyle patterns.

	Low-PRS (n = 14,420)	Medium-PRS (n = 21,641)	High-PRS (n = 4201)	Gene-Nutrient Interaction *p* Value
Low energy ^1^ High energy	1	1.771 (1.505–2.084) 2.237 (1.661–3.012)	2.960 (2.503–3.502) 3.592 (2.650 4.870	0.0002
Low KBD ^2^ High KBD	1	1.857 (1.611–2.141) 1.961 (1.636–2.351)	3.005 (2.597–3.477) 3.220 (2.674 3.878)	0.6555
Low PBD ^2^ High PBD	1	1.891 (1.638–2.183) 1.917 (1.621–2.266)	2.903 (2.453–3.508) 3.130 (2.700 3.627)	0.3048
Low WSD ^2^ High WSD	1	1.891 (1.638–2.183) 1.963 (1.632–2.361)	3.130 (2.700–3.627) 3.329 (2.754 4.023)	0.0347
Low RMD ^2^ High RMD	1	1.891 (1.638–2.183) 1.995 (1.677–2.374)	3.130 (2.700–3.627) 3.221 (2.694 3.850)	0.0715
Low alcohol ^3^ High alcohol	1	1.867 (1.548–2.253) 1.937 (1.550–2.421)	3.048 (2.513–3.697) 3.254 (2.588 4.093)	0.3049
Low exercise ^4^ High exercise	1	2.229 (1.758–2.827) 1.711 (1.428–2.049)	3.667 (2.874–4.679) 2.845 (2.363 3.426)	0.4115
Non-smoking + former smoking Smoking	1	1.835 (1.580–2.131) 3.021 (1.979–4.611)	3.068 (2.631–3.577) 4.889 (3.154– 7.576)	0.0006

Values represent adjusted odd ratios and 95% confidence intervals. Covariates included age, sex, education, income, energy intake (percentage of estimated energy requirement), residence areas, daily activity, alcohol intake, and smoking status. PRS with 8 SNPs of the best GMDR model was divided into three categories according to the risk alleles: when the number of risk alleles in the PRS was ≤3, 4–5, and ≥6 into Low-PRS, Middle-PRS, and High-PRS, respectively. Reference was the low-PRS. ^1^ <Estimated energy requirement defined in dietary reference index; ^2^ <75th percentiles; <20 g/day; ^3^ <20 g daily alcohol intake; ^4^ <moderate exercise for 150 min/day.

## Data Availability

The raw data involved in this study will be available by the authors to any qualified researcher.

## Supplementary Materials

The following supporting information can be downloaded at: https://www.mdpi.com/article/10.3390/nu14153222/s1, Table S1: Characteristics of all genetic variants selected from GWAS for type 2 diabetes at *p* < 5 × 10^−6^.
