# Supplementary material for: Association of Polygenic Variants with Type 2 Diabetes Risk and Their Interaction with Lifestyles in Asians
Source: Nutrients. 2022 Aug 6;14(15):3222. doi: 10.3390/nu14153222 (PMC9370736; doi:10.3390/nu14153222)
Supplement: Supplementary file 1 [file nutrients-14-03222-s001.zip › nutrients-1831761-supplementary.pdf]

Table S1. Characteristics of all genetic variants selected from GWAS for type 2 diabetes at $P < 5 \times 10^{-6}$.

CHR	SNP	BP	A1	A2	OR	SE	P	Gene names	location	MAF	HWE
2	rs72976740	161299818	T	C	1.311	0.05724	2.19×10^{-6}	RBMS1	Intron	0.03336	0.2494
3	rs73134196	13276044	G	A	0.8266	0.03248	4.52×10^{-9}	IQSEC1	Intron	0.1575	0.4186
3	rs7631705	23632234	C	T	0.8884	0.02424	8.00×10^{-9}	UBE2E2	3_prime_utr	0.3341	0.5715
3	rs113386200	63832212	C	T	0.8894	0.02294	3.29×10^{-7}	THOC7	Intron	0.4183	0.5814
6	rs35612982	20682622	C	T	1.342	0.02259	9.35×10^{-39}	CDKAL1	Intron	0.4649	0.1131
7	rs2191349	15064309	G	T	0.8906	0.02427	2.91×10^{-7}	DGKB	Non-coding transcript	0.3233	0.04656
7	rs61160304	127249659	T	C	1.492	0.03797	6.34×10^{-26}	PAX4	Non-coding transcript	0.07384	0.2274
8	rs72664959	69363625	T	C	1.207	0.03917	1.58×10^{-6}	C8orf34	Nmd transcript	0.08153	0.07773
8	rs13266634	118184783	T	C	0.8529	0.02329	8.22×10^{-12}	SLC30A8	Missense	0.398	0.9656
9	rs7034200	4289050	A	C	1.113	0.0227	2.05×10^{-7}	GLIS3	Nmd transcript	0.4064	0.3467
9	rs10811661	22134094	C	T	0.7965	0.02306	6.33×10^{-24}	CDKN2A/B	Non-coding transcript	0.4387	0.1998
10	rs12764758	94516663	T	C	1.285	0.04837	5.00×10^{-10}	IDE	Non-coding transcript	0.04856	0.3958
11	rs60808706	2857233	A	G	0.7874	0.02367	6.65×10^{-25}	KCNQ1	Intron	0.3913	0.2251
11	rs757110	17418477	C	A	1.126	0.02277	1.84×10^{-7}	ABCC8	Missense	0.4012	0.6183
11	rs36036422	120476821	T	C	0.7579	0.06021	4.16×10^{-6}	GRIK4	Intron	0.04462	0.175
12	rs11414025	66225188	GT	G	1.116	0.02348	2.80×10^{-6}	HMGA2	Nmd transcript	0.3409	0.8615
15	rs11853287	77399371	G	A	1.113	0.02262	2.15×10^{-6}	PEAK1	3_prime_utr	0.4248	0.8262
17	rs11651052	36102381	A	G	1.157	0.02406	5.17×10^{-10}	HNF1B	Intron	0.3009	0.4383
20	rs3859609	42823424	T	C	0.8935	0.02312	1.11×10^{-6}	OSER1	Non-coding transcript	0.3988	0.4185
20	rs6103720	43010130	G	A	0.8762	0.02851	3.55×10^{-6}	HNF4A	Intron	0.2069	0.1219
